# Minnesota wild hemp: a crucial botanical source in early cannabinoid discovery

**DOI:** 10.1186/s42238-020-00031-3

**Published:** 2020-09-11

**Authors:** Crist N. Filer

**Affiliations:** 1 PerkinElmer Health Sciences Inc., 940 Winter Street, Waltham, MA 02451 USA; 2 549 Albany St., Boston, MA 02118 USA

**Keywords:** Cannabinoid, Federal Bureau of Narcotics, Minnesota wild hemp, Roger Adams, University of Illinois

## Abstract

Renewed and sustained *Cannabis* chemistry exploration was initiated by Roger Adams at the University of Illinois Chemistry Department with cooperation from the Treasury Department Narcotics Laboratory in the early 1940’s. This partnership and time investment by both parties made practical sense. Adams was able to explore natural products chemistry and the Narcotics Laboratory began to clarify the chemistry mysteries of *Cannabis*. Minnesota wild hemp, often viewed as just a roadside weed, was employed as the critical botanical source. Based on its widespread cultivation during World War II, this was also a very pragmatic decision. Although the unique Illinois – Washington D. C. collaboration lasted only a few short years (1939–1942), the stunning results included the isolation and extensive characterization of cannabidiol, the structure elucidation and total synthesis of cannabinol as well as the identification of the tetrahydrocannabinol structure as an intoxicating pharmacophore. Furthermore, this research well prepared many junior chemists for prolific careers in both academia as well as industry, inspired the discoveries of later *Cannabis* investigators and also provided a successful model of a productive academic-government partnership.

## Background

Human cultivation of the genus *Cannabis* and exploitation of its useful materials easily dates back several thousand years (Russo 2007). Despite this long common history, it is only in the last 80 years that significant progress been made in understanding the fascinating details of *Cannabis* chemistry. *Cannabis* is the source of hundreds of varied natural products and it is reported that 177 of these are the polyketide-terpene hybrids known collectively as cannabinoids (Hanus et al. 2016). Natural product isolation and characterization is an especially challenging process involving human creativity along with available resources. Further complicating progress in *Cannabis* science by the mid-twentieth century was that the attitude in the United States toward *Cannabis* was quickly changing. Once viewed in the late nineteenth century as a fashionable panacea, by the early 1940’s *Cannabis* was under increased scrutiny and near prohibition (Bridgeman and Abazia 2017). It would take a strong and disciplined personality to navigate these changing regulatory issues and advance *Cannabis* science. Equally interestingly was the fact that a feral variety of *Cannabis*, Minnesota wild hemp, emerged as the critical botanical source for the profound discoveries in early cannabinoid chemistry.

### History

The first isolation and characterization studies in the cannabinoid area began in England with the work of Wood, Spivey and Easterfield in 1896 using *Cannabis indica* (Wood et al. 1896). As would be the case for decades of future *Cannabis* research, obtaining lipophilic cannabinoids as crystalline solids from natural sources was a frustrating challenge. However, these workers eventually purified a substance as its acetate which upon hydrolysis gave a phenolic compound that they named cannabinol. As so often happened in this natural products area, ongoing progress in cannabinoid research was then abruptly stopped for decades. In the early 1930’s, Robert S. Cahn again resumed work on cannabinol, isolating it from the same earlier source *Cannabis indica*. Cahn also prepared cannabinol derivatives, testing them with color forming reactions and culminating in a close yet incorrect structure prediction for cannabinol (Cahn 1933). However, sustained research and success on cannabinol and other cannabinoids waited for almost another decade and a unique *Cannabis* source as well as the creativity of an academic natural products chemist whose reputation was already gaining momentum.

Harvard trained organic chemist Roger Adams began his extraordinary career at the University of Illinois Chemistry Department in 1916. By 1939 Adams was already in mid-career, directing an extremely active group of a dozen or so graduate students producing a steady stream of seminal papers in prestigious publications like the *Journal of the American Chemical Society.* Regarding natural products, Adams fearlessly took on more challenging projects with the high risk of failure but also potentially large reward. Many of these studies had important agricultural benefits. Relevant for this discussion, in 1939 Adams started another marathon natural products project which changed *Cannabis* chemistry forever.

In 1939, Adams was approached by the Treasury Department Bureau of Narcotics (today’s equivalent is the Drug Enforcement Administration) with an interesting proposition; namely, to carefully examine the still largely unknown chemistry of *Cannabis*. Almost certainly, Adams was selected because of his celebrated success in natural products and this proposal would have clearly appealed to him for several reasons. First, the briefly explored *Cannabis* chemistry had been all but abandoned years before and there were still many intriguing and unanswered questions. Secondly, the Treasury Department provided Adams with permission to work in this increasingly government supervised area. At that time, *Cannabis* was heavily regulated by the Marijuana Tax Act (MTA) of 1937 which did not prohibit it but still imposed expensive fines and possible imprisonment for violations, thereby strongly discouraging *Cannabis* use (Pisanti and Bifulco 2017). Finally, the Narcotics Laboratory offered to provide Adams with all the *Cannabis* raw material that he would need. Specifically, Minnesota wild hemp (*Cannabis sativa*) was chosen as the botanical source for this renewed *Cannabis* research. Also known as “Ditch Weed,” this plant had been well described in an earlier classic botanical treatise (Oswald and Boss 1918). This annual seed propagated *Cannabis* variety known for its strong fiber content grew freely without cultivation along roads and rivers in Minnesota. Curiously, Oswald and Boss regarded this wild hemp as only an obnoxious weed, third on their list of several dozen botanical offenders and they provided specific guidance about its eradication. However, Minnesota wild hemp was a very practical plant choice for Adams’ research at that time. With World War II looming and access to valuable fibers from the Pacific rim curtailed, the government was actively growing large amounts of this hardy fiber producing hemp in midwestern states including Minnesota.

The details regarding the Minnesota wild hemp harvesting and initial large-scale processing are technically fascinating and partially outlined below, excerpted from the Experimental section of Adams’ first cannabinoid paper (Adams et al. 1940f):

“The hemp used in these experiments grew wild in Minnesota during the season of 1938. It was cut in August, after flowering had begun but before seed had “set” in the female tops. It was stored for 6 weeks in a room where a fan assured circulation of air in order to dry it completely. No molding occurred. The material was then beaten and shaken to remove the course stems which amounted to about one third of the total dry weight. The stems were discarded and the relatively fine material that remained was extracted with 95% ethanol …”.

The above procedure was performed at the Treasury Department Narcotics Laboratory in Washington D. C. on a 40 kg hemp batch along with its large volume ethanol extraction process. The ethanol extracts were then concentrated to a small volume by an elaborate distillation system followed by petroleum ether dilution, several water washes and final distillation to yield about 100 g of a purified red oil. This red oil, so frequently referred to in Adams’ many subsequent papers, was supplied to his University of Illinois laboratory. While the Minnesota wild hemp extraction operation was ongoing, Adams no doubt was pouring over the earlier Robert S. Cahn 1930’s publications as a valuable resource for developing his own technical strategy of cannabinoid isolation from the Minnesota wild hemp red oil. Based on that research, Adams was very aware there was little chance of directly crystallizing low melting cannabinoids themselves from the red oil but often, higher melting derivatives of them could be. Here is where Adams first encountered an immediate and annoying technical problem. In contrast to the earlier results of Cahn, Adams could not obtain a crystalline cannabinoid acetate or *p*-nitrobenzoate derivative from the Minnesota wild hemp red oil. Devoting only one dispassionate sentence of his first paper to this inconvenient setback, Adams was not terribly surprised about the outcome. Cahn had utilized a distinctly different *Cannabis* source for his earlier work, containing either different cannabinoids or differing amounts of them. This technical reversal challenged Adams’ creativity, but it also provided him with a wonderful chance for invention.

**Scheme 1:**
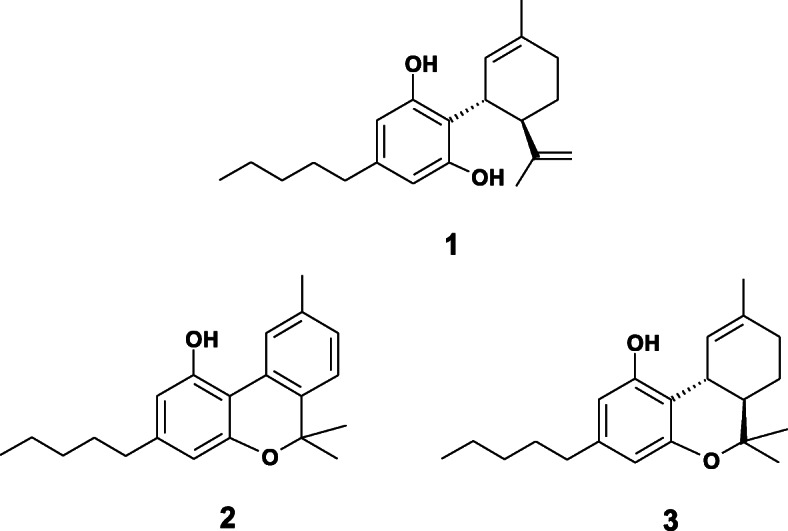
Structures of cannabinoids referred to: cannabidiol (**1**), cannabinol (**2**) and delta-9-tetrahydrocannabinol (**3**)

Adams’ account does not indicate the number of any failed attempts, but he finally reported success in isolating a significant amount of a bis-3, 5-dinitrobenzoate cannabinoid derivative from the Minnesota wild hemp red oil. Gentle ambient temperature ammonolysis of this substance with ammonia in toluene and typical organic reaction workup afforded a pure compound as a pale yellow resin which Adams named cannabidiol. He later developed an improved cannabidiol bis-3, 5-dinitrobenzoate derivatization procedure “where 45-50% of the purified red oil can be shown to be cannabidiol” (Adams et al. 1940e) and was also awarded a US patent (Adams 1942) for his novel process. As sometimes happens in chemistry, the initial failure to reproduce the earlier literature result was truly an inventive opportunity for Adams. Using all the chemistry and biology tools available to him in 1940, Adams characterized cannabidiol as thoroughly as possible, noting that it was non-intoxicating (Russo 2017) as well as decorated with an asymmetric carbon framework as evidenced by its significant negative optical rotation. Unfortunately, this stereochemistry complexity confounded Adams’ ability to derive a completely accurate structure for cannabidiol (Adams et al. 1940a). This frustrating structure conundrum for cannabidiol took another two decades to ultimately resolve as structure **1** (Scheme 1) by the essentially simultaneous discovery of Mechoulam (Mechoulam and Shvo 1963) and Santavy (Santavy 1964). As a precursor to this achievement, in his paper Mechoulam credited Adams’ “masterly investigations” and “substantial progress” in the *Cannabis* field. However, Adams’ exploration of the Minnesota wild hemp red oil continued and proved to be even more rewarding. He was also able to isolate from it both the known inositol derivative quebrachitol as well as the earlier reported cannabinol whose structure Adams conclusively proved to be **2** (Scheme 1) by unambiguous total synthesis (Adams et al. 1940d). Remarkably, the most extraordinary cannabinoid chemistry discovery by Adams in this already productive Minnesota wild hemp exploration was yet to come.

Based on the above results, Adams had essentially isolated all the major constituents from this bountiful midwestern botanical source. However, Adams was soon to discover at the bench a most amazing reaction involving cannabidiol. Although he was not able to decipher the complete structure of cannabidiol, Adams did establish that it contained an isopropenyl moiety proximate to several resorcinol hydroxyl groups. Treating cannabidiol with a number of different acidic reagents, Adams was easily able to force one of the resorcinol hydroxyls to ring close on the nearby isopropenyl tertiary carbon position, forming several optically active cyclized products, each with a dimethylpyran ring analogous to that of cannabinol (Adams et al. 1940c). Adams simply described these products as putative tetrahydrocannabinols. Having nearly the same complicated carbon skeleton motif as cannabidiol, Adams was again not able to completely deduce the exact structure of these tetrahydrocannabinols but believed that they were very likely olefin isomers of each other. Furthermore, these cyclized products were found to be intoxicating. Adams also prophetically suggested that some tetrahydrocannabinol would eventually be found naturally in *Cannabis* itself (Adams et al. 1940b). Two decades later in 1964, Mechoulam (Gaoni and Mechoulam 1964), isolated the physiologically active (intoxicating) delta-9-tetrahydrocannabinol (**3,** Scheme 1) from *Cannabis,* confirming the exact structure proposed by Santavy (Santavy 1964).

## Discussion

The year 1940 was a watershed moment for *Cannabis* chemistry. The United States Treasury Department Bureau of Narcotics, tasked with the challenging goal of protecting Americans from the medical and economic harm of addictive substances, acted under the auspices of the 1937 MTA. State and federal laws regarding *Cannabis* were then more aligned in discouraging *Cannabis* use than now. In fact, after enactment of the MTA in 1937 all states made possession of *Cannabis* illegal (Sacco 2014) although the exact timing of each state’s ban of *Cannabis* after 1937, especially Illinois, is unclear. Despite the increasingly prohibitive climate toward *Cannabis* in 1940, an interesting collaboration was forged then between the Treasury Department Narcotics Laboratory and Roger Adams at the University of Illinois. Given the functional prohibition of *Cannabis* medical use by the MTA, it is interesting to consider why the Treasury Department approached Adams to explore *Cannabis* chemistry? The answer may well be in examining the contrasting social and scientific status of *Cannabis* compounds with opiates. Although opiate addiction was then perceived as a greater problem than *Cannabis* by the Treasury Department, the structure of morphine (Gulland and Robinson 1923) and its analogues along with the advancing knowledge of opiate pharmacology had already been worked out years before. In contrast, less problematic *Cannabis* with its constituent compounds and their pharmacology was still largely a mystery for both government and science. No literature discussion was located regarding the ease with which Adams could have obtained his own *Cannabis* raw material. However, even if he could, the generous supply of finely processed Minnesota wild hemp red oil from the Treasury Department was likely too hard for Adams to resist. In this mutually beneficial partnership, Adams eagerly explored some fascinating natural products chemistry while the Narcotics Laboratory increased its technical understanding of *Cannabis* and potentially the identification of its physiologically active compounds*.* The Narcotics Laboratory’s use of Minnesota wild hemp as the botanical resource was a practical decision based on its widespread midwestern cultivation during World War II.

By the end of 1940, momentous achievements were rapidly accomplished in this fruitful chemistry collaboration: The new cannabinoid, cannabidiol, was isolated and extensively characterized. The structure and total synthesis of cannabinol was finally and unequivocally established. Several tetrahydrocannabinols were also identified and found to be pharmacologically intoxicating. Although the structures of cannabidiol and the tetrahydrocannabinols were not completely elucidated, Adams’ discoveries strongly influenced the thinking and contributions of future chemists such as Raphael Mechoulam. The extensive SciFinder® database reveals that Adams’ early cannabinoid chemistry continues to be favorably cited, especially by many current *Cannabis* authors (Tarragon and Moreno 2019). Specifically, an analysis of the 16 papers produced by the Adams laboratory in 1940 revealed that only two were uncited. The remaining papers were cited a total of 167 times and all but 4 of those citations occurred in 2000 or later. Adams’ chemistry achievements were recognized by numerous awards including the coveted Priestley Medal of the American Chemical Society. While no single award can be attributed to Adams’ contributions in *Cannabis* alone, the cannabinoid area was a significant part of his larger body of work and prize-winning chemistry.

The prolific work in the Adams’ laboratory also proved to be a valuable incubator for the young scientists involved. Even though it appears that none of Adams’ students ever worked again on *Cannabis* chemistry, their intense technical training during that period was extremely useful for their varied careers in academia and industry. Although there is no indication that the University of Illinois efforts directly influenced *Cannabis* regulations, the Narcotics Laboratory was well pleased with the Adams’ laboratory findings in their report for 1940, noting the same significant technical results mentioned above (Anonymous 1940). Also, during this time period of increasing concern over *Cannabis,* this academic-government collaboration demonstrated that the plant could contain components without the drawback of abuse. In particular, the interesting discovery by Adams that cannabidiol was non-intoxicating provided the initial evidence that a cannabinoid might even be a potential drug candidate. Ironically, the rapid progress and quick succession of achievements early in this unique and productive partnership also brought it to a rather quick conclusion with the last collaborative paper between them appearing in 1942 (Adams et al. 1942). Adams published his final independent paper in the *Cannabis* area in 1949 (Adams et al. 1949), moving on to other intriguing topics and further chemistry acclaim.

As for Minnesota wild hemp, in the early 1940’s it clearly performed a critical role not only as a crucial fiber plant for World War II but also as a key botanical source of profound *Cannabis* chemistry discoveries. Although Minnesota wild hemp has now quietly returned to the original humble status assigned by Oswald and Boss in 1918, its legacy of chemistry contributions will remain a noteworthy matter of *Cannabis* history.

## Data Availability

Not Applicable.

## Abbreviations

DEA: Drug Enforcement Administration
NMR: Nuclear Magnetic Resonance
MTA: Marijuana Tax Act of 1937
